# Evidence of myomiR regulation of the pentose phosphate pathway during mechanical load‐induced hypertrophy

**DOI:** 10.14814/phy2.15137

**Published:** 2021-12-09

**Authors:** Taylor Valentino, Vandre C. Figueiredo, C. Brooks Mobley, John J. McCarthy, Ivan J. Vechetti

**Affiliations:** ^1^ Department of Physiology College of Medicine Lexington Kentucky USA; ^2^ Center for Muscle Biology University of Kentucky Lexington Kentucky USA; ^3^ Department of Physical Therapy College of Health Sciences University of Kentucky Lexington Kentucky USA; ^4^ School of Kinesiology Auburn University Auburn Alabama USA; ^5^ Department of Nutrition and Health Sciences College of Education and Human Sciences University of Nebraska‐Lincoln Lincoln Nebraska USA

**Keywords:** myomiR, NADPH, pentose phosphate pathway, redox metabolism, skeletal muscle hypertrophy

## Abstract

Many of the molecular and cellular mechanisms discovered to regulate skeletal muscle hypertrophy were first identified using the rodent synergist ablation model. This model reveals the intrinsic capability and necessary pathways of skeletal muscle growth in response to mechanical overload (MOV). Reminiscent of the rapid cellular growth observed with cancer, we hypothesized that in response to MOV, skeletal muscle would undergo metabolic programming to sustain increased demands to support hypertrophy. To test this hypothesis, we analyzed the gene expression of specific metabolic pathways taken from transcriptomic microarray data of a MOV time course. We found an upregulation of genes involved in the oxidative branch of the pentose phosphate pathways (PPP) and mitochondrial branch of the folate cycle suggesting an increase in the production of NADPH. In addition, we sought to determine the potential role of skeletal muscle‐enriched microRNA (myomiRs) and satellite cells in the regulation of the metabolic pathways that changed during MOV. We observed an inverse pattern in gene expression between muscle‐enriched myomiR‐1 and its known target gene glucose‐6‐phosphate dehydrogenase, *G6pdx*, suggesting myomiR regulation of PPP activation in response to MOV. Satellite cell fusion had a significant but modest impact on PPP gene expression. These transcriptomic findings suggest the robust muscle hypertrophy induced by MOV requires enhanced redox metabolism via PPP production of NADPH which is potentially regulated by a myomiR network.

## Graphical abstract

During rest, myomiR‐1 levels are high resulting in the repression of Glucose‐6‐Phosphate Dehydrogenase (G6pdx). Mechanical overload (MOV) results in the decrease of myomiR‐1, and enhanced levels of G6pdx causing an increase in the activation of the pentose phosphate pathways (PPP). Delivery of a satellite cell‐enriched miR‐206 upon fusion of satellite cells at the late phase of MOV, targets G6pdx. In the absences of satellite cell fusion, G6pdx levels remain higher resulting in sustained activation of the PPP.

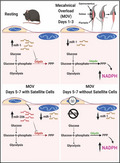

## 1 INTRODUCTION

First described almost 60 years ago, the rodent synergist ablation model has been instrumental in identifying many of the cellular and molecular mechanisms involved in the regulation of skeletal muscle hypertrophy which were subsequently shown to be conserved in humans in response to resistance exercise (Baar & Esser, 1999; Bodine et al., 2001; DeVol et al., 1990; Goldberg et al., 1975; Hornberger et al., 2006). In particular, synergist ablation has been reported to increase mouse plantaris muscle weight by 30%–50% following 14 days of MOV (Roberts et al., 2020; Terena et al., 2017; Vechetti et al., 2019). The supra‐physiological growth induced by synergist ablation‐induced MOV reveals the remarkable intrinsic capability of skeletal muscle to hypertrophy. The identification of the metabolic processes that allow for such rapid and robust hypertrophic growth may provide insight that can be used to enhance the recovery of muscle following periods of disuse or under conditions in which the growth response to a hypertrophic stimulus is impaired such as with aging.

Metabolism has an essential role during cellular growth, whereby multiple pathways generate and provide the necessary requirements needed for anabolism (Zhu & Thompson, 2019). However, despite this evidence, during skeletal muscle hypertrophy the focus has primarily been on mTOR signaling, energy systems that rapidly produced ATP such as glycolysis and the phosphocreatine pathway, and the energy sensor AMPK (Egan & Zierath, 2013; Hargreaves & Spriet, 2020; Kjobsted et al., 2018; Miyazaki & Esser, 2009). The role of metabolic reprograming during skeletal muscle growth has only recently been investigated. For example, it was demonstrated the participation of important metabolic processes, such as the polyamine pathway, hexosamine biosynthetic pathway, and serine synthesis pathways play a role in skeletal muscle hypertrophy (Lambert et al., 2018; Stadhouders et al., 2020; Tabbaa et al., 2021). Therefore, overlooked metabolic networks may be contributing to the regulation of muscle mass.

MOV induces rapid and robust skeletal muscle growth which resembles the accelerated growth that occurs in cancer cells. It has been demonstrated that to be able to handle the high rates of growth, cancer cells alter their metabolism to rely more heavily upon the fermentation of glucose to lactate despite the presence of oxygen and functioning mitochondria (Hsu & Sabatini, 2008; Liberti & Locasale, 2016; Sun et al., 2018; Warburg et al., 1927). This metabolic phenomenon known as the Warburg Effect (first described by Otto Warburg) was initially thought to be critical for supplying ATP at a high rate necessary to support the rapid cell growth associated with cancer (Liberti & Locasale, 2016). This perspective has shifted in recent years to one in which the main purpose of aerobic glycolysis (Warburg Effect) is not ATP production per se but rather the generation of glycolytic intermediates that serve as precursors for the macromolecules (e.g., nucleotides, amino acid, and lipids) required for cell growth (Rosenzweig et al., 2018). In addition, metabolic reprograming could be a way in which redirecting metabolism modulates metabolic intermediates that subsequently dictates cellular behavior. This is seen in the control of epigenetics (Diehl & Muir, 2020), transcription factors (Li et al., 2018; Lu et al., 2002), enzymes (Maguire et al., 2021), and increases in pathways which generate necessary substrates needed to fuel tumor growth (Fan et al., 2019). Together, these results suggest that metabolic reprogramming can induce a variety of cellular changes that could modify crucial signaling pathways related to growth. Since MOV‐induced skeletal muscle hypertrophy stimulates a supra‐physiological growth, we hypothesize that, similarly to the cancer cells, skeletal muscle fibers utilize aerobic glycolysis to support this rapid increase in size.

As an initial effort to test this hypothesis, we analyzed a microarray transcriptome time course analysis of MOV‐induced muscle hypertrophy and focused on genes specifically associated glucose metabolism. In support of our hypothesis, we show the upregulation of genes known to have a central role in the pentose phosphate pathway (PPP) and one‐carbon metabolism for the production of NADPH. We provide evidence for a role of muscle‐enriched myomiR‐1 in the regulation of the PPP by targeting glucose‐6‐phosphate dehydrogenase, *G6pdx*, a known target gene of myomiR‐1, and the rate limiting step of the PPP. Finally, we observed a modest response of PPP gene expression upon satellite cell fusion, providing evidence of the satellite‐enriched myomiR‐206 as an additional regulator of skeletal muscle metabolism during MOV. The findings from our bioinformatics analyses identified candidate genes that warrant further investigation and their respective role in the metabolic programming that permits the robust hypertrophic growth induced by MOV.

## 2 METHODS

### 2.1 Animals

All experimental procedures involving mice were approved by the University of Kentucky Institutional Animal Care and Use Committee. To deplete satellite cells, we crossed Pax7*^CreER^* ^/^ *^CreER^* mouse (stock no. 017763) to the Rosa26*^DTA^* ^/^ *^DTA^* mouse (stock no. 010527) (The Jackson Laboratory) to generate the Pax7‐DTA mouse as previously described by us (Fry et al., 2014; McCarthy et al., 2011; Murphy et al., 2011). Mice were housed in a temperature and humidity‐controlled room and maintained on a 14:10‐h light‐dark cycle with food and water ad libitum.

### 2.2 Experimental design

Adult (5 months old) female Pax7‐DTA mice were randomly assigned to receive either an intraperitoneal injection of tamoxifen (2.5 mg/day) or vehicle (15% ethanol in sunflower seed oil) for five consecutive days followed by a 2‐week washout period. Following the washout period, vehicle‐ and tamoxifen‐treated mice were then randomly assigned to sham or a synergist ablation surgery group with the plantaris muscle collected after 1, 3, 5, or 7 days (*n* = 5–6/group).

### 2.3 Synergist ablation

To induce skeletal muscle hypertrophy of plantaris muscle via mechanical overload (MOV), a bilateral synergist ablation surgery which results in significant skeletal muscle hypertrophy, was performed as previously described by our laboratory (Chaillou et al., 2013; Figueiredo et al., 2019; Hamilton et al., 2014; McCarthy & Esser, 2007; Vechetti et al., 2019). Mice were anesthetized (3% isoflurane with 1.5 L of O_2_ per minute), placed in sternal recumbence where a small incision was made on the dorsal aspect of the lower hind limb with approximately half of the gastrocnemius and entire soleus muscle carefully excised. No apparent damage was observed to the neural and vascular supply of the plantaris muscle following the surgery. At the designated time point post‐surgery, mice were euthanized via CO_2_ inhalation followed by cervical dislocation with the plantaris muscles excised, weighed, snap frozen in liquid nitrogen, and stored at −80°C until downstream analyses.

### 2.4 RNA isolation

Total RNA was isolated from plantaris muscle previously frozen in liquid nitrogen. Samples were homogenized using a tissue homogenizer (Bullet Blender, Next Advance Inc.). Following homogenization, RNA was isolated via phase separation by the addition of bromochloropropane (BCP) and centrifugation at 15,000 × *g* for 15 min. The supernatant containing RNA was then washed with the assistance of the Direct‐zol™ Kit (Zymo Research). RNA was treated in‐column with DNAse and eluted in nuclease‐free water. The total RNA concentration and purity were assessed by measuring optical density (230, 260, and 280 nm) with a Nanodrop™ 2000/2000cSpectrophotometer (ThermoFisher Scientific).

### 2.5 Microarray dataset analysis

The microarray analysis was carried out with pooled samples as previously described by us (Chaillou et al., 2013). Briefly, 250 ng of total RNA from a pool of 2 or 3 animals (same amount of total RNA), resulting in *n* = 2 per group, was used for each time point. The RNA was loaded onto the GeneChip™ Mouse Gene 1.0 ST array (Affymetrix). This microarray dataset is a part of another study by our laboratory (currently in preparation for submission) and has been deposited in the NCBI Gene Expression Omnibus database (GSE153542). Microarray analysis focused only on genes that are involved in metabolic pathways. Bioinformatics analyses of the dataset were performed in R (version 4.0.2) utilizing *Bioconductor* packages. Briefly, raw signal intensity data were downloaded and normalized with robust multi‐array average (RMA) using GEOquery (Davis & Meltzer, 2007) and oligo (Carvalho & Irizarry, 2010) packages, respectively. Normalization consisted in background correction, quantile normalization, and log2 transformation. For the detection of differentially expressed genes (DEG) related to metabolic pathways, we initially utilized microarray data from vehicle‐treated mice to determine changes associated with the rapid growth induced by MOV. Having identified DEGs from vehicle‐treated mice, we then adjusted our statistical design to include microarray data from tamoxifen‐treated mice to determine if satellite cell depletion affected the change in metabolic‐related gene expression identified in vehicle‐treated mice in response to MOV. To identify DEGs, a linear model was fitted to the normalized data using Linear Models for Microarray Data (Limma) package (Ritchie et al., 2015). For vehicle‐treated mice only analysis, DEGs were estimated using the empirical Bayes function with a false discovery rate (FDR) 5%, using the Benjamini–Hochberg method. For comparison between conditions (vehicle‐treated vs. tamoxifen‐treated), DEGs were detected using a group‐mean parametrization with *p* < 0.05 due to the small sample size of *n* = 2 (pooled samples).

### 2.6 MicroRNA expression

Reverse transcription reactions for myomiR‐206 and U6 small nuclear RNA (Rnu6) were performed with 10 ng of total RNA using Taqman MicroRNA Reverse Transcription Kit (ThermoFisher Scientific) according to the manufacturer's directions. qPCR was carried out with Taqman Gene expression Master Mix (2×) (ThermoFisher Scientific), TaqMan gene expression assay (miR‐206, #000510; Rnu6, #001973) using cDNA in a 10 µl reaction volume. qPCR was performed using the QuantStudio3 (Applied Biosystems) qPCR system as described by the manufacturer. qPCR efficiency was calculated by linear regression during the exponential phase using LinRegPCR software v11.126 (Ruijter et al., 2009). The comparison of miRNAs expression between groups was determined following normalization with Rnu6. Relative quantification of miRNA expression was assessed by the ΔΔCT method relative to the control.

### 2.7 In silico prediction of miRNA target genes

Target genes were detected using miRanda (Enright et al., 2003) and RNAhybrid (Kruger & Rehmsmeier, 2006) software. Since individual tools use different features for miRNA:target interactions, we utilized a custom Python script to select only shared miRNA:target gene between the two software programs with a minimal free energy set as Δ*G*°= −18 kJ.

### 2.8 Statistical analysis

The data are presented as means ± standard error. Except for transcriptomic data (statistical analyses discussed above), two‐way ANOVA with Tukey post hoc analysis was used to test the effect of genotype and intervention on microRNA levels. Statistical analysis was performed with GraphPad Prism version 9.2.0 (GraphPad Software). Statistical significance was set at *p* < 0.05.

## 3 RESULTS

### 3.1 Glycolytic pathway

**FIGURE 1:**
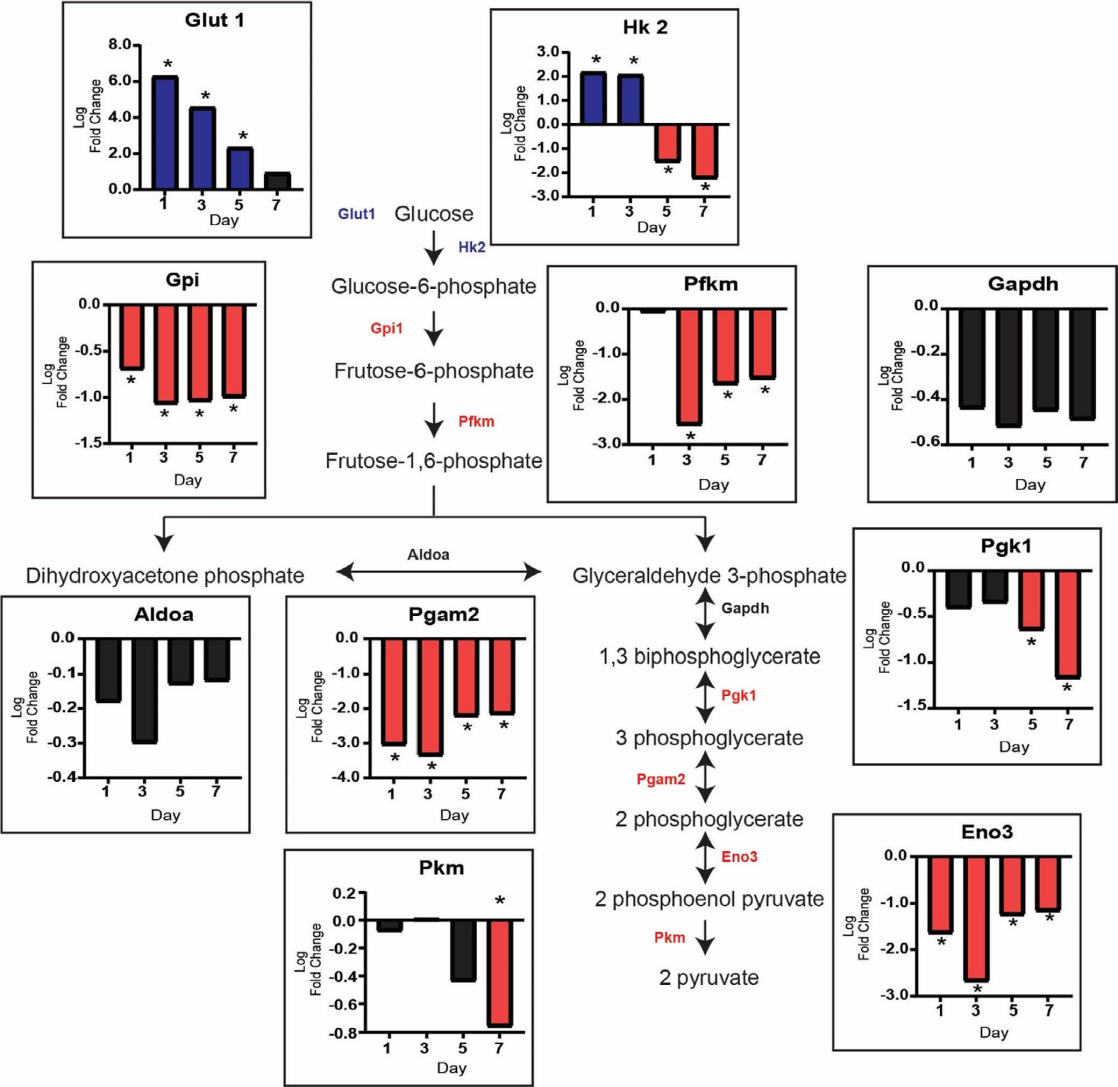
Glucose uptake does not drive glycolysis during the rapid growth induced by mechanical overload (MOV). The glycolytic pathway is not utilized during MOV as demonstrated by the log twofold change (blue: upregulated, red: downregulated and black: not differentially expressed) in expression of the genes involved in glycolysis. *N* = 2/group (pooled samples). *denotes statistical significance (adjusted *p* value <0.05) compared to sham (control)

To determine if aerobic glycolysis might be involved in providing the precursors for the macromolecules necessary for the growth induced by MOV, we analyzed a microarray time course of MOV‐induced muscle hypertrophy for genes known to be involved in metabolic pathways (Figure S1). Starting with glycolysis, we observed higher levels of genes involved with glucose uptake and intracellular phosphorylation (*Glut1* and *Hk2*, respectively) in response to MOV (Figure 1). *Glut1* expression was higher throughout the time course, returning to baseline by day 7, whereas *Hk2* level was elevated on days 1 and 3 but lower on days 5 and 7 of MOV. Downstream of *Hk2*, the level of the remaining genes involved in the glycolytic pathway was either unchanged (*Gapdh* or *Aldoa*) or lower (*Gpi*, *Pfkm*, *Pgk1*, *Pgam2*, *Pkm*, and *Eno3*) at one or more time points during MOV (Figure 1). The change in the levels of glycolytic genes suggested that glucose were being diverted to adjacent central carbon pathways.

### 3.2 Pentose phosphate pathway (PPP)

**FIGURE 2:**
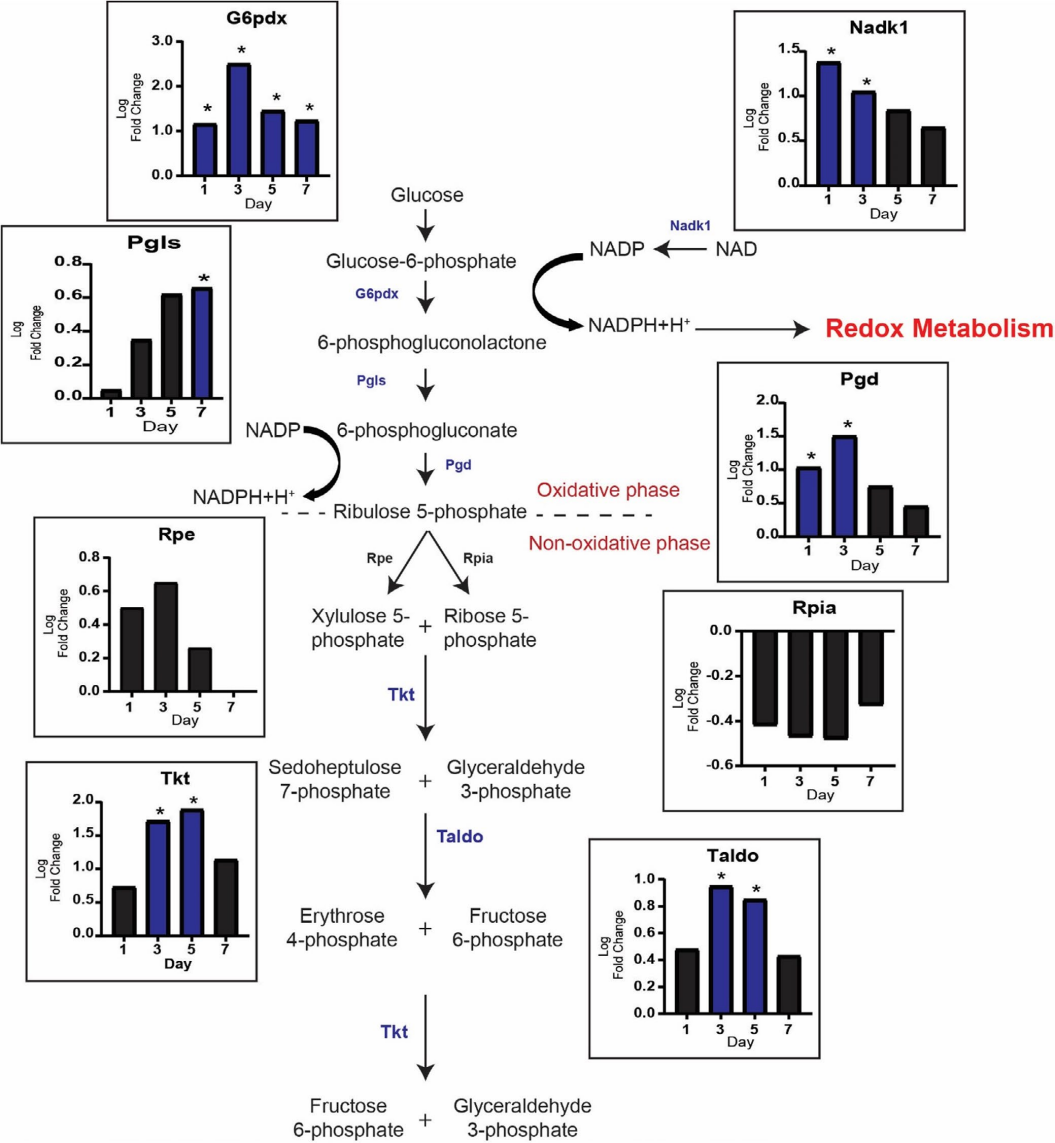
Pentose Phosphate Pathway (PPP) is diverted from glycolysis during mechanical overload (MOV). PPP and Redox metabolism are upregulated during MOV as highlighted by log twofold change (blue: upregulated and black: not differentially expressed) of genes involved in these reactions. *N* = 2/group (pooled samples). *denotes statistical significance (adjusted *p* value <0.05) compared to sham (control)

To determine if the PPP was activated during MOV‐induced muscle hypertrophy, we analyzed genes of both the oxidative and the non‐oxidative branches of the PPP. In the oxidative branch, the rate limiting enzyme *G6pdx* gene level was higher throughout the time course with the downstream genes *Pgls* and *Pgd* elevated on day 7 and days 1 and 3 of MOV, respectively (Figure 2). For the non‐oxidative branch, the gene level of *Rpe* and Rpia was not significantly different from sham whereas *Tkt* and *Taldo1* were higher on days 3 and 5 of MOV (Figure 2). The increase in expression of G6pdx could lead to a subsequent increase in NADPH production. NADPH is the reduced form of NADP^+^, therefore we sought to determine if *Nadk1* (the kinase that phosphorylates NAD^+^), could concomitantly increase as well; possibly as a means to provide more substrate for *G6pdx*. We determined that the expression of *Nadk1* was higher on days 1 and 3 of MOV (Figure 2).

### 3.3 One‐carbon metabolism

#### 3.3.1 Serine synthesis pathway (SSP)

**FIGURE 3:**
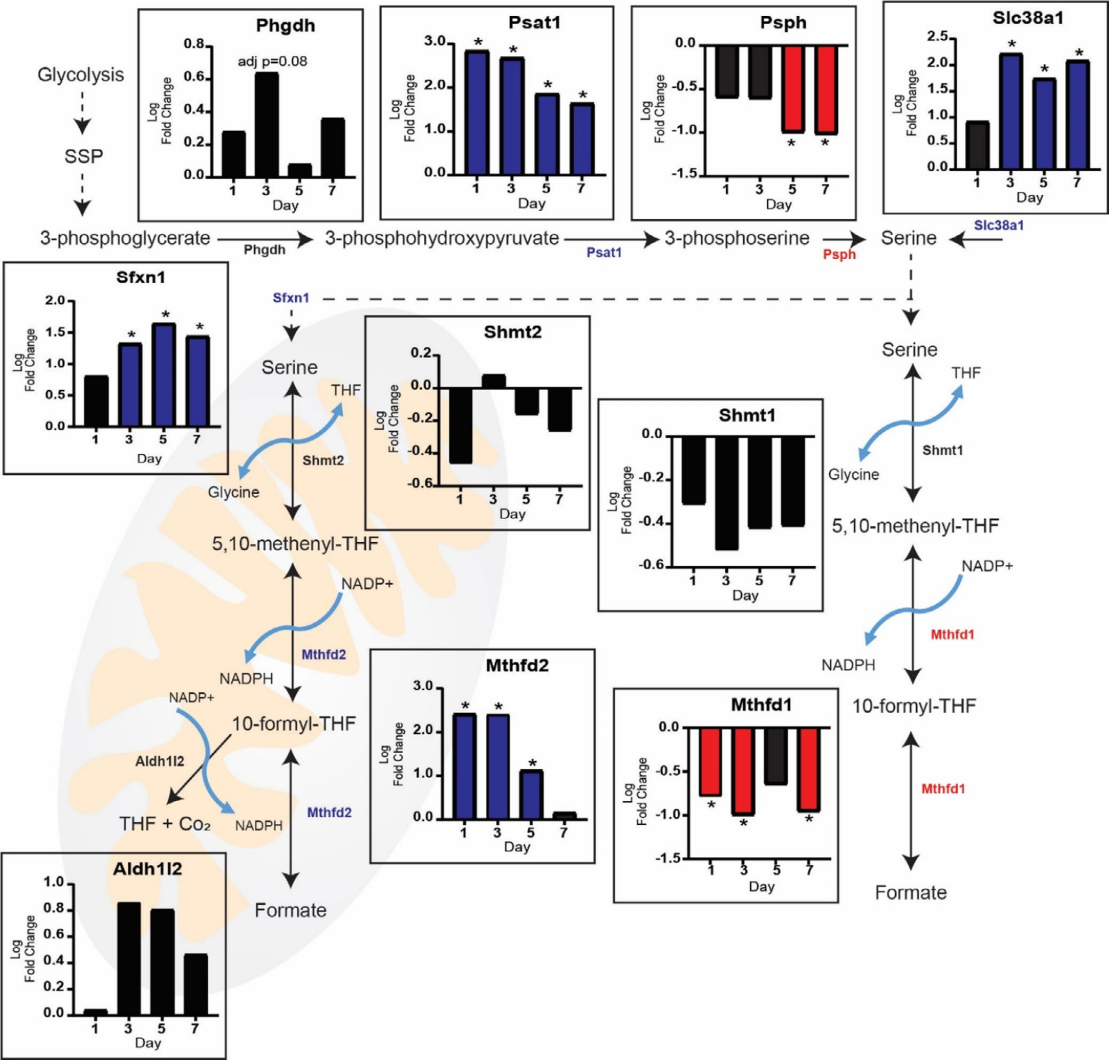
Serine and the folate cycle contribute additional NADPH during MOV: Serine may be generated via the serine synthesis pathway or brought into the cell by the sodium‐coupled neutral amino acids transporter (Slc38a1). Serine can then participate in the generation of NADPH though the folate cycle. The mitochondrial compartment of the folate cycle is upregulated during MOV as highlighted by log twofold change (blue: upregulated and black: not differentially expressed) of genes involved in these reactions. *N* = 2/group (pooled samples). *denotes statistical significance (adjusted *p* value <0.05) compared to sham (control)

The higher level of the genes associated with the oxidative branch of the PPP and *Nadk1* suggested the production of NADPH was enhanced rather than de novo nucleotide synthesis in response to MOV. This finding motivated us to investigate if the levels of one‐carbon metabolism genes involved in NADPH production and nucleotide synthesis (Newman & Maddocks, 2017) were also higher during MOV. We first looked at the serine synthesis pathway (SSP), as serine is used to generate the folate intermediate 5,10‐methylenetetrahydrofolate, which participates in the production nucleotides and NADPH (Geeraerts et al., 2021). We observed the level of serine synthesis pathway (SSP) genes which feeds serine into the folate cycle to generate NADPH, was altered with MOV. As shown in Figure 3, the lack of a pattern in the expression of SSP genes *Phdgh*, *Psat1*, and *Psph*, suggested the de novo synthesis of serine was not elevated in response to MOV. However, level of the sodium‐coupled neutral amino acid transporter, *Slc38a1*, was higher on days 3, 5, and 7 of MOV suggesting extracellular serine could be the source needed for downstream central carbon pathways (Figure 3).

#### 3.3.2 The folate cycle

The folate cycle is compartmentalized in the cytosol and mitochondria in which parallel reactions support one‐carbon anabolic pathways (Ducker & Rabinowitz, 2017). The level of cytosolic *Mthfd1* gene was lower throughout the time course suggesting the cytosolic compartment of the folate cycle was not participating in NADPH production during MOV (Figure 3). In contrast, the level of mitochondrial *Mthfd2* gene was highly elevated on days 1 and 3 of MOV suggesting high levels of NADPH could be produced via the mitochondrial folate cycle (Figure 3). Furthermore, we found that the gene which encodes for a mitochondrial serine transporter, *Sfnx1*, was higher on days 3, 5, and 7 of MOV. This supports the elevation in *Slc38a1* which could bring extracellular serine into the cell which further gets pumped into the mitochondria for utilization of the folate pathway.

In terms of pathways related to reductive biosynthesis, we did not observe any significant change in the levels of genes involved in either fatty acid or amino acid synthesis, suggesting that these pathways were not altered at the transcript level in response to MOV (Figure S1). For nucleotide synthesis, the lack of a consistent pattern in the gene expression in the non‐oxidative branch of the PPP, made it difficult to determine if there was a change in de novo nucleotide synthesis. However, there was higher levels of genes (*Aprt*, *Nme*, and *Hprt*) of the purine salvage pathway across the MOV time course (Figure S2), suggesting the participation of the folate cycle in purine synthesis. Additionally, *Cmpk1*, *Rrm2*, *Ctps*, *Tyms*, and *Uck1* were elevated at some time point during MOV (Figure S3). Finally, the elevated level of the neutral amino acid transporter *Slc38a10* on days 3, 5, and 7 of MOV may enhance pyrimidine synthesis by increasing intracellular glutamine and aspartate levels (Figure S3).

### 3.4 Redox metabolism

**FIGURE 4:**
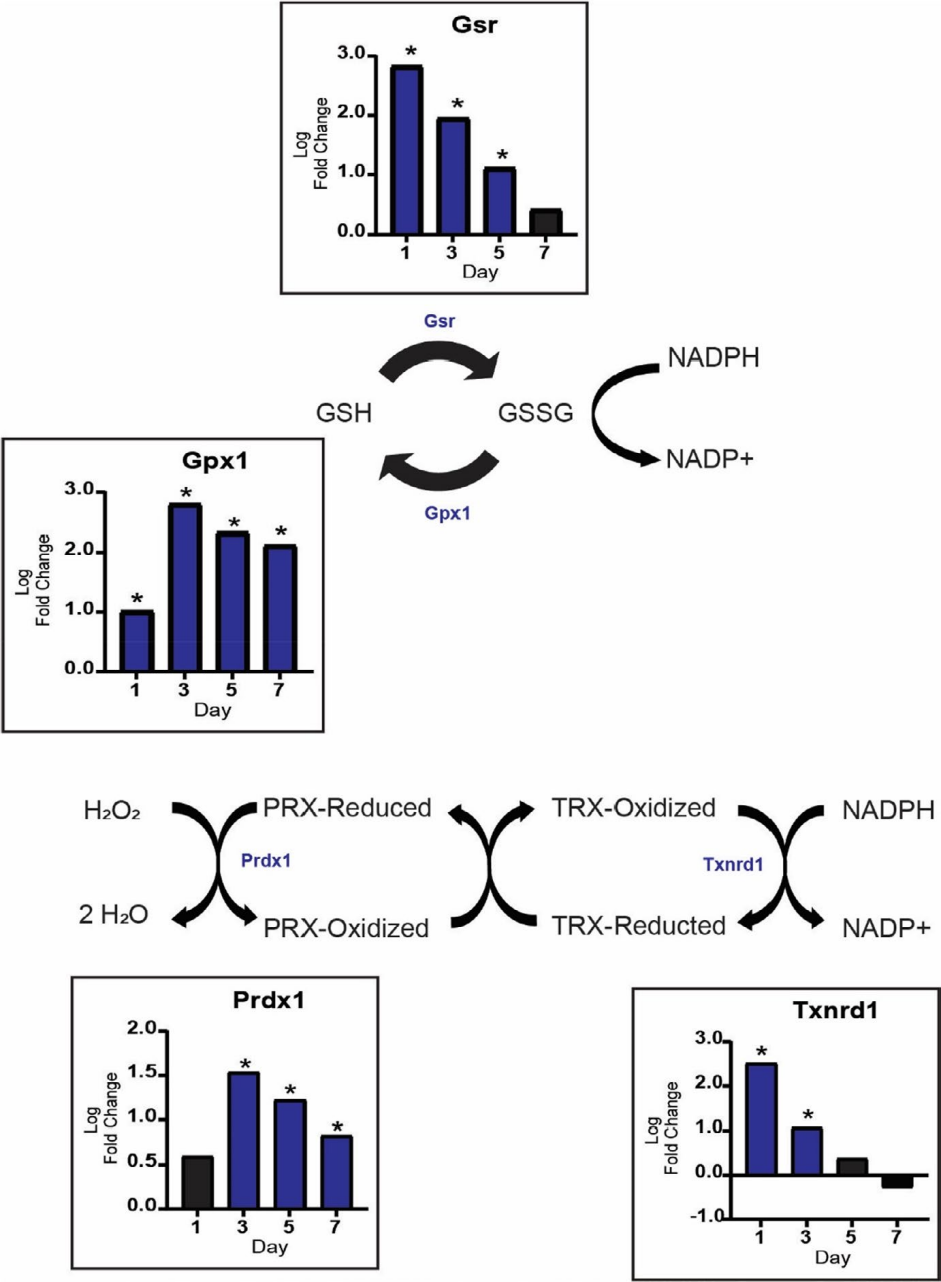
Redox metabolism is increased during MOV. Pathways related to the oxidation and reduction of glutathione are upregulated during MOV. In addition, other pathways that utilize NADPH to scavenge hydrogen peroxide increase during the first week of MOV as highlighted by log twofold change (blue: upregulated and black: not differentially expressed) of genes involved in these reactions. *N* = 2/group (pooled samples). *denotes statistical significance (adjusted *p* value <0.05) compared to sham (control)

The elevated levels of genes of both the oxidative branch of the PPP and the mitochondrial folate cycle suggested higher NADPH synthesis in response to MOV. NADPH is used in redox homeostasis and reductive biosynthesis to produce fatty acids, amino acids, and nucleotides. NADPH plays a major role in cellular redox homeostasis by combating oxidative stress through reducing glutathione, thioredoxins, and peroxiredoxins. In response to MOV, *Gsr*, and *Gpx1*, which encode the two enzymes directly involved in glutathione reduction and oxidation, respectively, showed higher levels and suggest redox homeostasis was likely the primary process consuming NADPH (Figure 4). In addition, genes for thioredoxins and peroxiredoxins (*Txnrd1* and *Prdx 1*, *respectively*) involved in other redox pathways which can utilize NADPH to control hydrogen peroxide (H_2_O_2_) (Netto & Antunes, 2016) were also highly elevated during MOV (Figure 4).

### 3.5 Potential regulation of metabolic genes by myomiR‐1 during MOV

**FIGURE 5:**
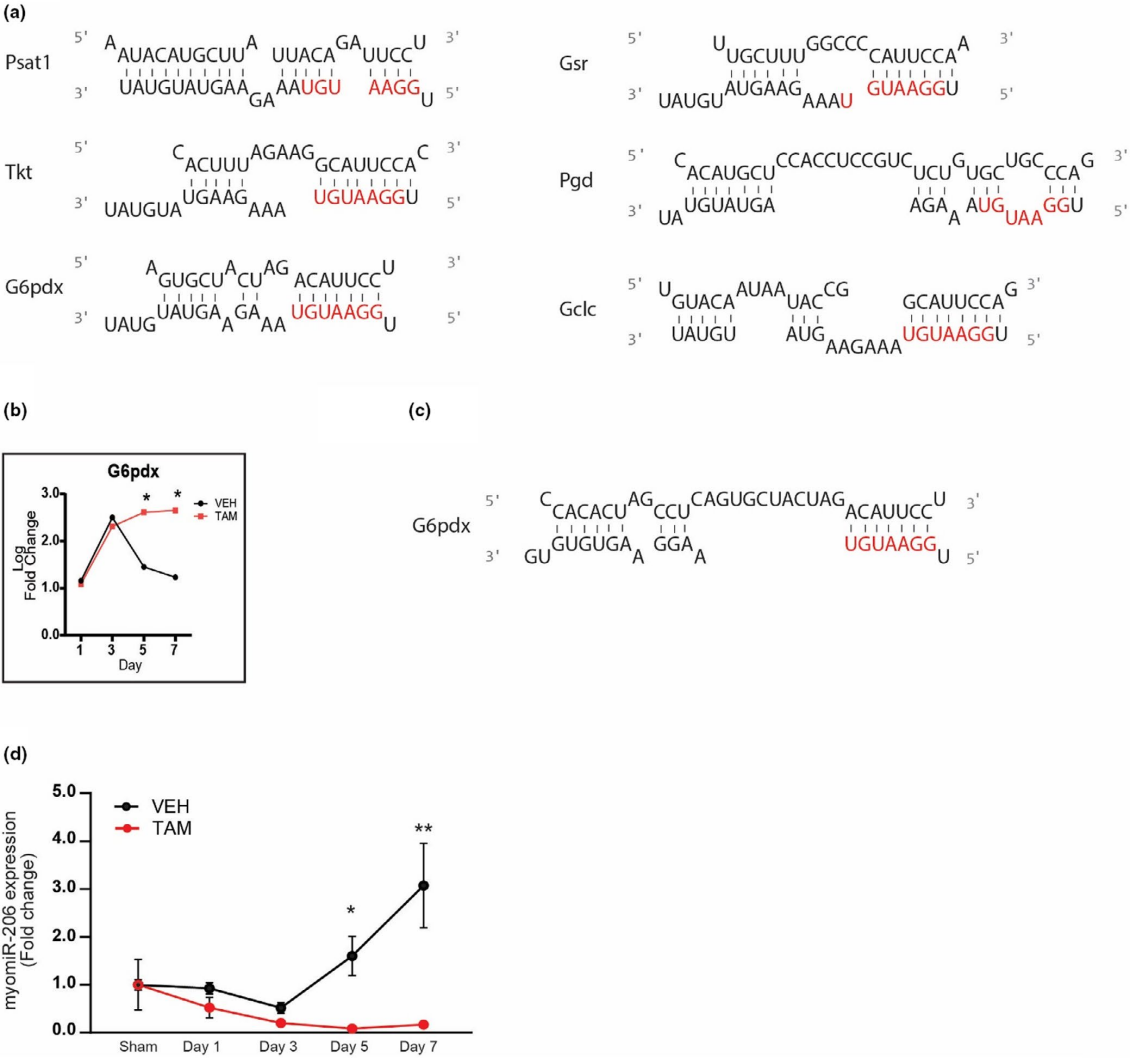
Skeletal muscle metabolism regulated by a myomiR‐1 network. (a) In‐silico prediction of myomiR‐1 metabolic target genes 3’ UTR seed sequence and miyomiR‐1 (b) Expression of G6pdx in SC+ and SC‐ skeletal muscle throughout the time course (c) G6pdx 3'‐UTR seed sequence and myomiR‐206 (d) myomiR expression throughout the time course. Log twofold change of DEGs between Vehicle‐ and Tamoxifen‐treated mice after mechanical overload. *N* = 2/group (pooled samples) for (a‐c), and *n* = 6 for (d). *denotes statistical significance (*p* value <0.05) between vehicle‐ and tamoxifen‐treated mice

MyomiR‐1 is the most abundant miRNA in the mouse plantaris muscle and shows the greatest change in expression in response to MOV of any miRNA (Vechetti et al., 2021). To determine if the higher levels of PPP and redox‐related genes might be attributed to a decrease in myomiR‐1 expression, we performed a myomiR‐1 target gene prediction analysis on all of the significantly upregulated genes across the MOV time course. This analysis identified a small set of genes involved in the PPP, SSP, and redox homeostasis with predicted myomiR‐1 seed sequences within their respective 3'‐UTR (Figure 5a). In particular, genes of the oxidative (*G6pdx* and *Pgd*) and non‐oxidative (*Tkt*) branch of the PPP, SSP (*Psat1*), and redox homeostasis (*Gsr* and *Gclc*). Together, these results indicate a possible role of myomiR‐1 in the regulation of skeletal muscle metabolism by targeting key genes that act synergistically to produce and utilize NADPH during MOV.

### 3.6 Influence of satellite cells on metabolism during MOV

We next wanted to determine if satellite cell fusion might be affecting the expression of PPP and redox homeostasis‐related genes. We compared plantaris muscle gene expression of vehicle‐treated, satellite cell‐replete (SC+), and tamoxifen‐treated, satellite cell‐depleted (SC‐) to identify differentially expressed key metabolic genes across the MOV time course (Figure S4). Specifically, this analysis found that SC‐ *G6pdx* level was significantly higher on days 5 and 7 of MOV compared to SC+; in particular, SC‐ *G6pdx* level remained elevated while SC+G6pdx level was returning towards baseline on days 5 and 7 of MOV (Figure 5b).

Given that myomiR‐206 has been previously shown to be enriched in satellite cells (Fry et al., 2017), we hypothesized that the expression of myomiR‐206, which is known to target many of the same genes as myomiR‐1 given their identical seed sequence (Figure 5c), would be higher in SC+compared to SC‐ and, as a result, repress expression of these genes upon satellite fusion starting at day 5 of MOV. In Figure 5d, qPCR analysis confirmed miR‐206 expression was higher at days 5 and 7 of MOV in the SC+group whereas miR‐206 expression did not change across the MOV time course in SC‐ group, consistent with satellite cell proliferation and depletion, respectively.

## 4 DISCUSSION

We identified a potential mechanism which could facilitate the rapid growth response observed in MOV‐induced skeletal muscle hypertrophy. Specifically, we identified the pentose phosphate pathway (PPP) as a possible shunt for glucose and highlighted the importance of the rate limiting enzyme of the PPP, *G6pdx*, in the regulation of skeletal muscle metabolism during hypertrophy. In response to MOV, the results suggest the PPP becomes activated which leads to an increase in NADPH that is subsequently used in nucleotide biosynthesis and oxidative stress. We also provide evidence for a potential mechanism involving myomiRs modulating the expression of genes involved in the metabolic reprograming induced by MOV.

It is well established that the PPP is essential for reductive biosynthesis and generation of NADPH (Vander Heiden et al., 2009). Pharmacological inhibition of *G6pdx* reduced nucleotide synthesis and tumor cell proliferation in both pancreatic adenocarcinoma cells and mice hosting Ehrlich's ascitic tumor cells (Boros et al., 1997). In contrast, overexpression of *G6pdx* was shown to increase the levels of NADPH resulting in a higher rate of cellular growth under oxidative stress (Tian et al., 1998). While these studies were not conducted in skeletal muscle, they support a critical role of *G6pdx* in cell growth. The importance of the PPP in skeletal muscle adaptation has been recently described by Hoshino and colleagues whereby electrical stimulation of C2C12 myotubes led to an increase in reactive oxygen species‐dependent activation of the PPP (Hoshino et al., 2020). The reliance on the PPP for muscle growth is also supported by studies investigating the rapid muscle growth of broiler chickens and in mice upon inducible, muscle‐specific Akt‐activation (Abasht et al., 2016; Brothers et al., 2019). Finally, Wagner and colleagues found increased levels of *G6pdx* and *Pgd* in mice after Marcaine‐induced muscle damage indicating that the PPP is upregulated to assist in the recovery from muscle damage (Wagner et al., 1978). Together, these studies, along with the results of the current study, suggest that the PPP is responsive to various stimuli and highlights the potentially important role of this pathway in skeletal muscle hypertrophy and regeneration.

The PPP is not the only pathway which can generate NADPH, the folate and TCA cycles, malic enzymes, and nicotinamide nucleotide transhydrogenase mitochondrial inner membrane proteins can all generate NADPH (Ju et al., 2020). However, a previous study reported that in growing cells the PPP and folate cycle are the primary pathways responsible for NADPH production (Fan et al., 2014), which agrees with our transcriptomic data. In particular, the transcriptomic data provides evidence that the mitochondrial branch of the folate cycle, compared to the cytosolic branch, could generate additional NADPH during MOV (Figure 4). Ducker and co‐workers propose that the compartmentalization of the folate cycle is necessary in order to uncouple one‐carbon metabolism from glycolysis in an effort to preserve NAD^+^ and allow for the continuation of glycolysis (Ducker & Rabinowitz, 2017). Most recently Zhu and co‐workers determined that mitochondrial NADPH production was essential for the biosynthesis of proline, which contributed to collagen formation (Zhu et al., 2021). Therefore, these findings indicate that compartmentalizing NADPH synthesis could be a strategy by which the spatial production of a metabolic product ensures specific utilization.

It is well established that NADPH can be used to reduce oxidative stress with aberrant levels of NADPH associated with cancer, cardiovascular disease, and aging (Ju et al., 2020; Xiao et al., 2018; Ying, 2008). In skeletal muscle, it has been demonstrated that muscle contractions can increase both NADPH and ROS production (Henriquez‐Olguin et al., 2019; Hoshino et al., 2020). Interestingly, increased levels of ROS can hydroxylate guanines of mRNA (Kasai et al., 1991; Shan et al., 2007), decreasing translational fidelity, causing ribosomal stalling and shortened peptides (Shan et al., 2007; Simms et al., 2014; Tanaka et al., 2007). Furthermore, oxidative modifications can occur on ribosomal proteins and ribosome modifying enzymes which may interfere with translation (Shcherbik & Pestov, 2019). NADPH production could also attenuate errors in protein synthesis by reducing the abundance of free radicals that would otherwise cause expression of genes associated with DNA damage, oxidized mRNA, and proteolysis (Song et al., 2021). Altogether, increasing NADPH to reduce oxidative stress could allow for the high rate of protein synthesis that occurs during MOV and suggests that modulating NADPH could lead to improvements in protein synthesis.

Metabolic pathways are complex and regulated at several levels with microRNAs shown to be involved in the regulation of glycolysis and the PPP (Singh et al., 2013; Xu et al., 2017). In addition, we and others have previously demonstrated that microRNAs play a role in skeletal muscle plasticity (Bonanno et al., 2020; Hudson et al., 2014; Kovanda et al., 2018; McCarthy & Esser, 2007; Silva et al., 2019). For example, myomiR‐1, one of the most abundant microRNAs in skeletal muscle, decreases during skeletal muscle hypertrophy, suggesting this microRNA acts as a repressor of growth (Chaillou et al., 2013; McCarthy & Esser, 2007; Vechetti et al., 2021). Interestingly, myomiR‐1 has also been shown to repress the activity of *G6pdx* resulting in an inhibitory effect on the PPP (Singh et al., 2013). Our results propose that the rapid downregulation of myomiR‐1 in response to MOV leads to enhanced levels of *G6pdx* which, in turn, activates the PPP to generate NADPH. The concomitant increase in the levels of *G6pdx* with the decrease in myomiR‐1 may be the impetus for the metabolic shift observed during MOV.

It is well established that during MOV‐induced muscle hypertrophy, satellite cells fuse to the muscle fibers, contributing to the increase in myonuclei and potentially playing other hypertrophic supporting roles (Fry et al., 2014; Kirby et al., 2016; Snijders et al., 2015). In order to determine if satellite cell fusion affects the levels of *G6pdx*, during MOV‐induced muscle hypertrophy, we queried a microarray comparing the metabolic genes between SC+ and SC‐ mice. We found that *G6pdx* was significantly elevated in SC‐ when compared to SC+mice (Figure 5b). Previous results from our laboratory supported a mechanism in which the delivery of satellite cell‐enriched myomiR‐206‐induced collagen remodeling during MOV (Fry et al., 2017). In addition, myomiR‐206 has also been shown to regulate *G6pdx* in both C2C12 myoblasts and cancer cells (Jiang et al., 2019; Singh et al., 2013) as it shares a near identical seed region with myomiR‐1. The results from the current study demonstrate that myomiR‐206 expression is significantly elevated at days 5 and 7 of MOV only in SC+mice (Figure 5d) which coincided with the lower *G6pdx* expression in SC‐ muscle (Figure 5b). Together, these results suggest that satellite cell‐enriched myomiR‐206 also participates in the metabolic reprograming during MOV‐induced muscle hypertrophy by modulating the levels of *G6pdx*. Although our results are based on changes in gene expression and microRNA target prediction, the regulation of *G6pdx* expression by myomiRs has been validated in cancer and nonneoplastic fibroblast cells (Singh et al., 2013), where an inverse correlation between myomiR‐1, −206 and PPP gene expression was demonstrated. Thus, we propose that myomiR‐1 and myomiR‐206 contribute to the metabolic reprograming that allows for the robust skeletal muscle growth observed in response to MOV by activating the PPP and one‐carbon pathways.

The current study is not without limitations. The results rely exclusively on transcriptomic data to infer changes in metabolic pathways in response to MOV. A major focus of a future study will be to confirm the observed changes in gene expression are reflected at the protein level. Previous studies have reported that changes in metabolic gene transcript levels correspond to similar changes at the protein level (Gupte et al., 2011; Hoshino et al., 2020; Singh et al., 2013; Weyrauch et al., 2020). In addition, Weyrauch and colleagues, using the same hypertrophy model (i.e., MOV induced by synergist ablation) as used in the current study, showed that GLUT1 and G6PDH protein abundance as well as NADPH levels were all higher in response to 5 days of MOV (Weyrauch et al., 2020). This gives us confidence that the changes in gene expression observed here are also physiological meaningful. Additionally, samples were pooled for microarray analysis which limits the amount of biological variation in our results. This may limit the extent to which our results may be interpreted. Nonetheless, our results still provide novel data regarding metabolic networks that appear to be important for skeletal muscle growth and are not diminished by the methodology. For example, Mendias and co‐workers performed a microarray analysis on rat plantaris muscle after 3, 7, and 28s of synergist ablation (Mendias et al., 2017). RNA for this analysis was not pooled allowing for each sample (*n* = 4 per group) to be sequenced individually (Mendias et al., 2017). In line with our results, *G6pdx* was significantly higher on day 3 compared to control (GSE62388), demonstrating consistencies among microarray analysis strategies. Another potential limitation of the study is the fact that our transcriptomic analysis encompasses the whole muscle and cannot distinguish the contribution of non‐muscle cell types to the observed changes in gene expression. For instance, macrophage abundance has been shown be significantly elevated after 5 days of MOV which may have influenced the transcriptomic data given that macrophages undergo metabolic reprograming upon activation (Novak et al., 2009; O'Neill et al., 2016). This concern is tempered by the finding of Kirby and colleagues showing that approximately 90% of nascent RNA is associated with myonuclei during MOV induced by synergist ablation (Kirby et al., 2016), thus providing confidence that most of the observed changes in gene expression reflect what is occurring within muscle fibers.

In conclusion, we provide evidence to suggest the robust muscle growth induced by MOV may rely upon metabolic reprogramming that involves activation of the PPP and one‐carbon metabolic pathway. Further, our data along with studies in cancer, indicate activation of the PPP is regulated by myomiR targeting of *G6pdx* (Coda et al., 2015; Singh et al., 2013). The findings from this study provide the foundation for future studies focused on determining the role of *G6pdx* in skeletal muscle hypertrophy.

## CONFLICT OF INTEREST

The authors declare that there is no conflict of interest.

## AUTHOR CONTRIBUTIONS

Ivan J. Vechetti conceived and supervised the project. Taylor Valentino designed and performed the research. Vandre C Figueiredo performed the surgeries and RNA extraction. Taylor Valentino, John J. McCarthy, and Ivan J. Vechetti performed the data analysis. Taylor Valentino, C. Brooks Mobley, John J. McCarthy, and Ivan J. Vechetti interpreted the results. Taylor Valentino, John J. McCarthy, and Ivan J. Vechetti wrote the manuscript with input from all other authors. All authors agreed with the final version of the manuscript.

## Supporting information



Fig S1‐S4
