# Supplementary figures and images for: Evidence of myomiR regulation of the pentose phosphate pathway during mechanical load‐induced hypertrophy
Source: Physiol Rep. 2021 Dec 9;9(23):e15137. doi: 10.14814/phy2.15137 (PMC8661100; doi:10.14814/phy2.15137)

**Supplemenatry Figure 1.**


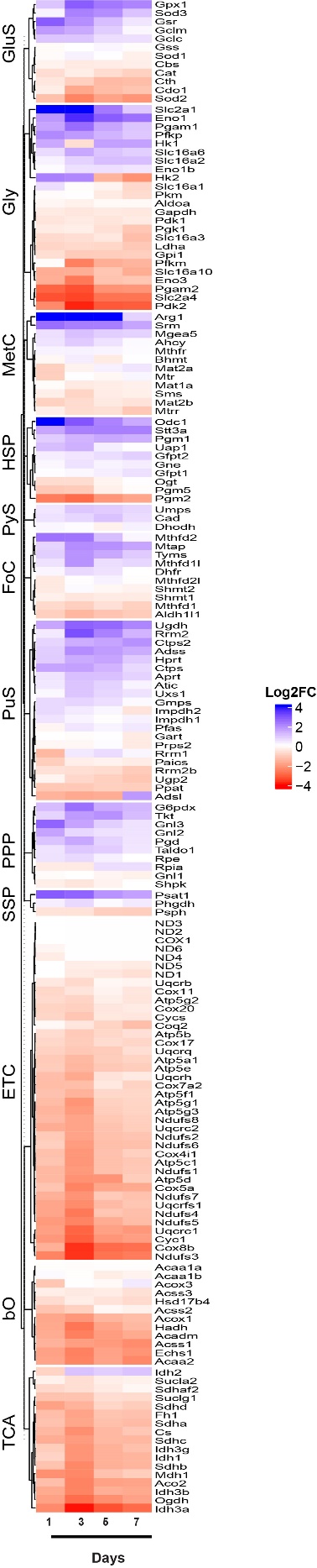


**Supplemenatry Figure 2.**

**
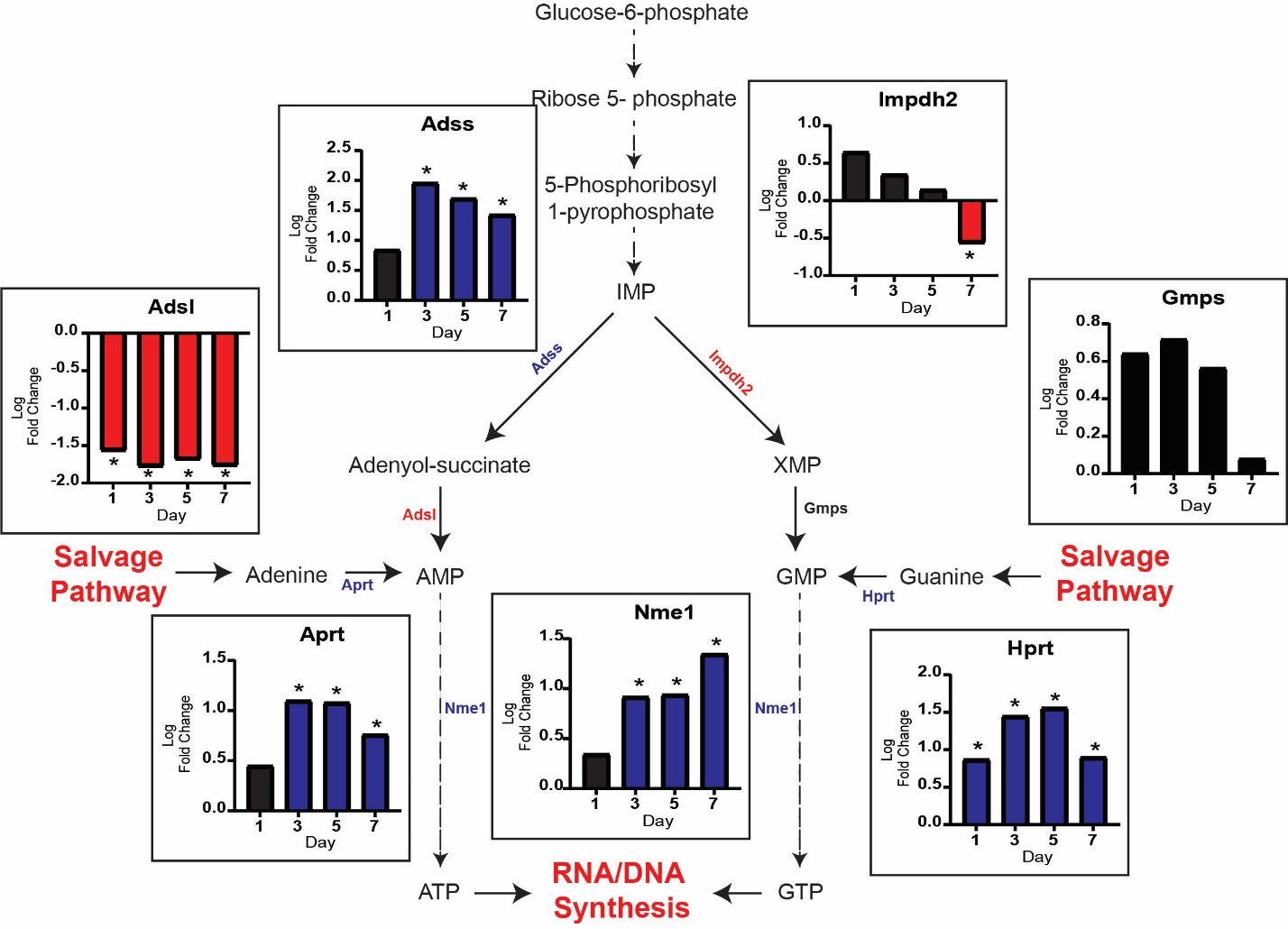
**

**Supplementary Figure. 3**

**
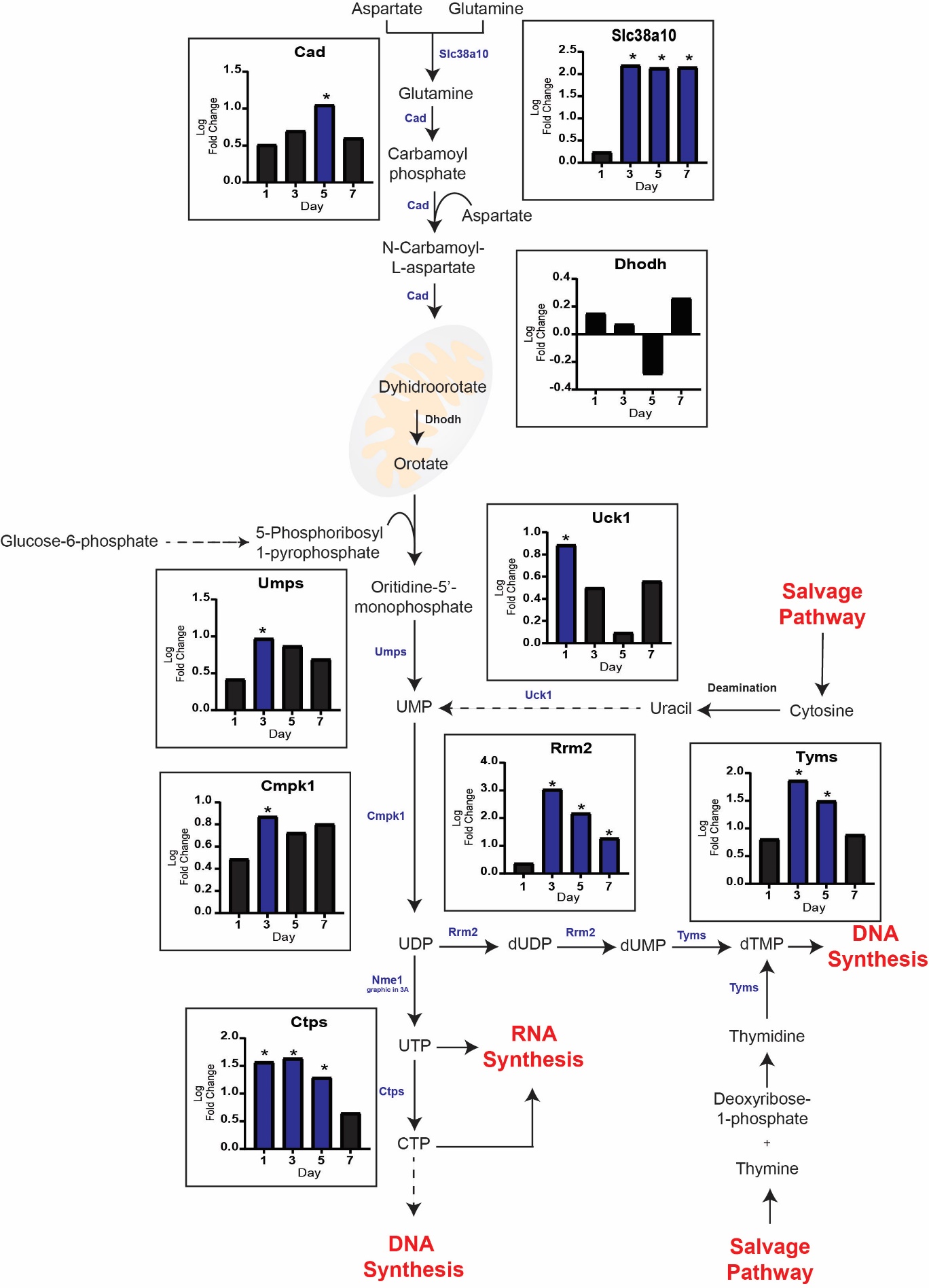
**

**Supplemenatry Figure 4.**

**
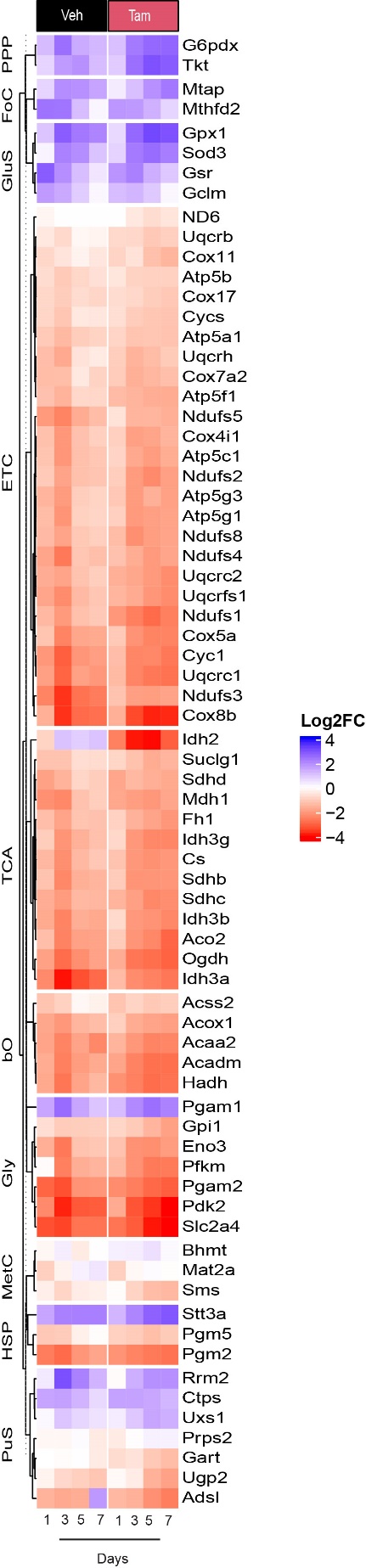
**

Supplement: Supplementary file 1 — Fig S1‐S4 [file PHY2-9-e15137-s001.docx]
